# Ultrasonography of the normal donkey tarsus (*equus asinus*)

**DOI:** 10.1038/s41598-024-61066-7

**Published:** 2024-05-07

**Authors:** Zakriya Almohamad

**Affiliations:** Department of Clinical Sciences, College of Veterinary Medicine, King Faisal University, PO Box 400, Al-Ahasa, 31982 Kingdom of Saudi Arabia

**Keywords:** Ultrasonography, Anatomy, Tarsus, Hock, Donkey, Anatomy, Medical research

## Abstract

Tarsal joint illness is a frequent source of hind limb lameness due to the complex anatomy of the region and the presence of numerous bony and soft tissue structures. Proper lameness diagnosis aims to discover the structure provoking lameness. Ultrasonography documents valuable information of soft tissues and characterizes soft tissue injuries that have heretofore been difficult to obtain either noninvasively or via radiography. The objectives of the current study were to develop and describe a standardized ultrasonographic protocol for investigation of the tarsal region in donkeys. The donkey tarsal anatomy was investigated in 5 cadavers and the tarsi of 11 healthy lameness free adult donkeys were echographically investigated. The dorsal, plantar, lateral and medial aspects of the tarsal region were substantially evaluated at four anatomical landmarks in both the longitudinal and horizontal planes using a multi-frequency 5–12 MHz linear transducer. Sonoanatomy of the extensor and flexor tarsal tendons, collateral and plantar ligaments, and synovial pouches was delineated and described. Systematic echography of the tarsal region allowed accurate localization and thorough exploration of various soft tissues of clinical interest in the donkey tarsus. Sonograms provided in this study should serve as a reference database for tarsal ultrasonography in clinical circumstances.

## Introduction

Donkey is an important member of equidae; however, little information is available about this creature, compared with horses^1^. Commonly, donkeys are treated like horses; despite of the anatomical, genetic and physiological differences^2^. Domestic donkeys tend to be classified by their size rather than breed into Miniature Mediterranean donkeys (36 inches or less), standard donkeys (36.01–48 inches), large standard donkeys (Jennets, 54 inches and Jacks, 56 inches) , and Mammoth Stock donkeys (over 56 inches)^3^. Donkeys have precious role in developing countries, particularly in rural zones, as a draught or riding animals and are often subjected to various injuries due to the nature of environment in such areas^4,5^. Consequently, musculoskeletal disorders and lameness are common sequellae^6^. Besides, lameness originating from the tarsal joint is a frequent presenting complaint of hind limb disorders^7^.

Physical examination is the first step for diagnosis of tarsal joint disorders. It involves various procedures including inspection and palpation of the area of interest to detect signs of inflammation, response to flexion and manipulation and the presence of effusion^8^. Swelling and effusion of the tarsocrural joint could be noticed easily; however, effusion of the inter-tarsal and tarsometatarsal joints can be difficult to appreciate via clinical examination alone^9^. In addition, distinction of the origin of effusion from the tarsal synovial structures or bursae is challenging, particularly in the presence of severe effusion or edema^10^. Accordingly, a thorough examination should be carried out to localize lameness emerging from the tarsal region^8^. Once the tarsus is determined as the source of lameness, further diagnostic imaging procedures would be needed to formulate a decisive diagnosis^11^. Radiography and ultrasonography are usually used for the initial assessment, as they are widely available, relatively inexpensive and do not require general anesthesia or specialized equipment^12^. Radiography is advocated for evaluation of bony structures; however, it is not particularly convenient for identification of soft tissue injuries and can be insensitive to early bone changes. When radiography fails to identify the specific problem or a soft tissue injury is suspected, ultrasonography is often employed^13^.

Ultrasonography has significantly improved the ability to confirm or rule out soft tissue disorders, which primarily involve the tarsal region^13^. Ultrasonography is noninvasive, widely available, and of comparable accuracy in the evaluation of tarsal joint pathologies^14^. Further merits of ultrasonography include portability, low cost, high spatial resolution, dynamic imaging, and could be used to guide various percutaneous interventions. Additionally, ultrasonography allows direct patient contact, facilitates immediate clinical correlation and provides the ability to compare with the contralateral region^15^.

Tarsal ultrasonography is usually requested in case of hock distention, wounds and/or lacerations, collateral ligament injury, and/or osteomyelitis in the vicinity of synovial structures^16,17^. Ultrasonography can localize the origin of tarsal distension through discriminating among cellulitis, synovial effusion, tendon injury and ligament damage. Besides, ultrasonography is well suited to detect penetration of wounds to the intra-articular structures and to evaluate the severity of soft tissue damage. Moreover, ultrasonography can distinguish the septic and non-septic arthritis and accurately assists aspiration of synovial fluid under ultrasound guidance for further analysis^18,19^. Arthritis is characterized on gray-scale ultrasound by mild anechoic synovial effusion and mildly thickened hyperechoic joint capsule. Sever synovial thickening and effusion, hypoechogenic to echogenic fibrinous loculations in conjunction with echogenic synovial fluid, are the typical ultrasonographic features of septic arthritis^19,20^.

The tarsus is a complex joint comprising numerous tendons and ligaments coursing in different directions and involves multiple synovial structures; therefore, it is susceptible to a considerable incidence of pathologies^21^. Successful ultrasound examination should be armed with a solid understanding of the normal tarsal anatomy^17^. Although, ultrasonography of the tarsal region has been previously studied in horses, camels and dogs^18,22–24^ , it has not been reported so far in donkeys. The objectives of the present study were to design and describe a standardized protocol for the ultrasonographic exploration of the hock joint and to afford reference images for ultrasonography of the tarsal region in sound donkeys.

## Results

Differentiation and demarcation of various soft tissue structures in the donkey tarsus was very well possible using the echographic imaging technology. Muscles, tendons as well as the muscle–tendon transition could be recognized and evaluated during their course over the hock joint.

### Dorsal tarsal ultrasonography

**Figure 1:**
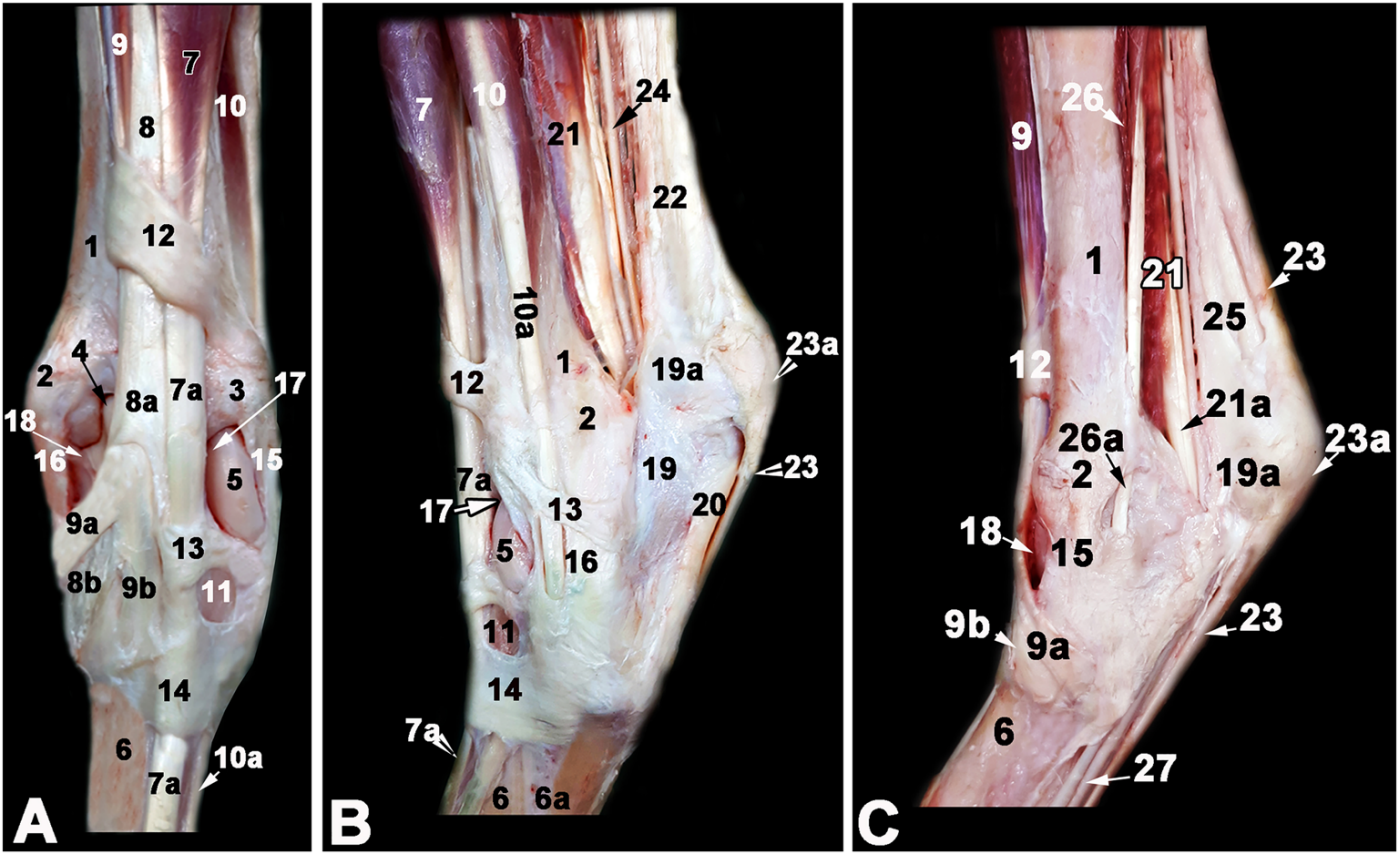
Gross anatomy of the tarsal region in donkey (**A**, dorsal left limb structures; **B**, lateral left limb structures; **C**, medial and plantar right limb structures). 1, tibia (body); 2, medial malleolus of the tibia; 3, lateral malleolus (distal part of the fibula); 4, medial trochlear ridge of the talus; 5, lateral trochlear ridge of the talus; 6, third metatarsal bone; 6a, fourth metatarsal bone; 7, long digital extensor muscle; 7b, long digital extensor tendon; 8, peroneus (fibularis) tertius muscle (fibrous muscle body); 8a, peroneus tertius opening of distal tendon for the tibialis cranialis tendon, 8b, metatarsal branch of peroneus tertius muscle; 9, tibialis cranialis muscle; 9a, tarsal (cunean) branch of tibialis cranialis muscle, 9b, metatarsal branch of tibialis cranialis muscle; 10, lateral digital extensor muscle; 10a, lateral digital extensor tendon; 11, short digital extensor muscle; 12, proximal (tibial) extensor retinaculum; 13, intermediate (tarsal) extensor retinaculum; 14, distal (metatarsal) extensor retinaculum; 15, medial collateral ligament; 16, lateral collateral ligament; 17, lateral pouch of the tarsocrural joint; 18, medial pouch of the tarsocrural joint; 19, calcaneus; 19a, tuber calcanei; 20, long plantar ligament; 21, lateral digital flexor muscle; 21a, tibialis caudalis, 22, common calcanean tendon;23, superficial digital flexor tendon; 23a, cap of the superficial digital flexor tendon; 24, tibial nerve; 25, gastrocnemius tendon; 26, medial digital flexor muscle; 27, deep digital flexor tendon.

**Figure 2:**
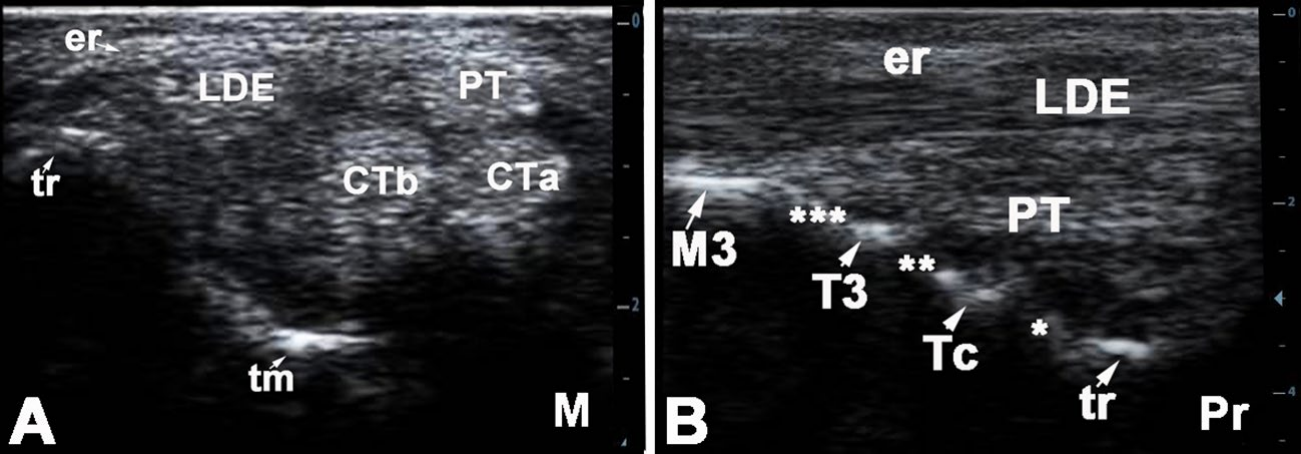
Dorsal transverse sonogram (**A**) at the level of the base of the calcaneus and dorsolateral longitudinal sonogram (**B**) of the normal left tarsal region in a 7 years old male donkey. M, medial; Pr, proximal; PT, peroneus tetius; CT, cranial tibial tendon; LDE, long digital extensor tendon; tm, medial trochlear ridge of the talus; tr, lateral trochlear ridge of the talus; er, Intermediate extensor retinaculum; CTa, cranial tibial tendon, medial branch; CTb, cranialt tibial tendon, metatarsal branch; tr, lateral trochlear ridge of the talus; Tc, central tarsal bon; T3, third tarsal bone; *, proximal intertarsal (talocentral) joint; **, distal intertarsal (centrodistal) joint; ***, tarsometatarsal joint.

On this side of the tarsus, tendons of the long digital extensor, fibularis (peroneus) tertius and cranial tibial muscles as well as the dorsomedial and dorsolateral tarsocrural joint pouches could be visualized on the transverse and longitudinal plane sonograms (Fig. 1A). In level l, the longitudinally directed and purely tendinous fibers of the fibularis tertius muscle tendon could be assessed as well as the belly of the cranial tibial muscle. Beneath the hypoechoic cranialis tibialis muscle, the highly reflective bone contour of the tibia could be seen. In level 2, the transition of the cranial tibial muscle into its end tendon could be recognized while forming a tunnel for the fibularis tertius to pass through and only a thin muscular tissue could be seen above the highly reflective arched contour of the talus (Fig. 2B).

In order to scan the long digital extensor tendon in the longitudinal plane, the transducer head was slightly moved and tilted about 15° laterally. The tendon sheath of the long digital extensor tendon was depicted as a thin anechoic line around the tendon. Applying a little pressure on the transducer permitted visualization of the cranial tibial vein that was recognized as a hypo-echoic to anechoic tube-shaped structure with hyperechoic margins. In level 3 between the talus and the central and third tarsal bones, the dorsolateral pouch of the tarsocrural joint could be depicted as hypoechoic to anechoic structure (Fig. 2).

In the horizontal plane, tendons of the long digital extensor and the fibularis tertius muscles could be evaluated in the same image. However, the exact demarcation between the fibularis tertius and tibialis cranialis muscles was vaguely possible in the sonograms of the first and second levels, since the fibularis tertius tendon perforated through the tendon of the tibialis cranialis muscle in this region (Fig. 2A).

In the fourth level, the long digital extensor tendon was seen lying directly on the main metatarsal bone. It could also be clearly defined further distally and traced to its junction with the lateral extensor tendon. The enveloping tendon sheath of the long digital extensor was still seen and appeared as a narrow anechoic area around the tendon.

**Figure 3:**
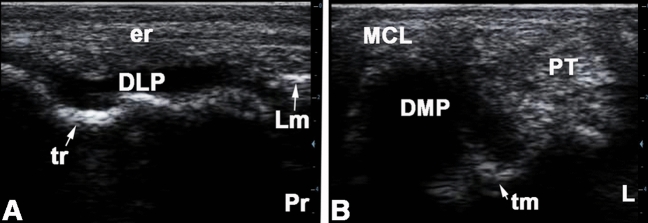
Longitudinal sonogram of the dorsolateral pouch (**A**) and transverse sonogram of the dorsomedial pouch of the tarsocrural joint of the normal right tarsal region in an 8 years old female donkey. Pr, proximal; L, lateral; Lm, lateral malleolus; DLP, dorsolateral pouch of the tarsocrural joint; er, intermediate extensor retinaculum; tr, lateral trochlear ridge of the talus; MCL, long medial collateral ligament; DMP, dorsomedial pouch of the tarsocrural joint; Pt, peroneus tertius tendon; tm, medial trochlear ridge of the talus.

A large sized medial pouch of the tarsocrural joint could be evaluated between the 2^nd^ and 3^rd^ levels by slightly moving the transducer head medial to the axial line of the joint and directing the beam lateroplantarly. With slight pressure, few amount of the anechoic synovial fluid was observed and could be differentiated from the hyperechoic contour of the talus. The hypoechoic synovial sac could be clearly separated from the surrounding tissues and deformed when the contact pressure was changed (Fig. 3B). The joint capsules of the proximal intertarsal, distal intertarsal, and tarsometatarsal joints were evaluated from the dorsum and appeared vaguely as a hypoechoic slit like structure between bone surfaces. No synovial fluid could be detected in those joints (Fig. 2B).

### Dorsolateral ultrasonography

In this area, the lateral digital extensor tendon and its sheath, the junction with the terminal tendon of the long digital extensor muscle and a small portion of the dorsolateral pouch of the tarsocrural joint could be evaluated (Fig. 1B).

In the longitudinal plane in level 1, the transition of the lateral digital extensor muscle into its terminal tendon could be observed. The musculature was characterized by delicate longitudinal echoes of its fibro-adipose muscle septa. In between there were the less echogenic muscle fibers. The parallel tendon fibers appeared as longitudinally oriented echogenic structures between the subcutis and bones of the tarsal joint. A hypoechoic to anechoic cavity could be seen lying directly on the bone, which corresponds to a portion of the tarsocrural joint pouch (Fig. 3A). In level 3, the lateral digital extensor tendon ran over the contour of the tarsal bones. In level 4, the distal retinaculum of the extensor tendons could be seen as a more echogenic, oval area between the subcutaneous tissue and tendon fibers in the sagittal section.

In the horizontal image at level 1, the tendon of the lateral digital extensor muscle could be recognized in the sulcus malleolaris lateralis with an ellipsoidal cross-section and homogeneous echogenic pattern. In level 2, the tendon became more rounded in shape. In level 3, the tendon had oval shape between the trochlea and the body of the talus and the base of the calcaneus. In level 4, the lateral digital extensor tendon was seen together with the long digital extensor tendon. Both tendons were seen between the subcutis and the main metatarsal bone and could be traced to their junction in the upper third of the metatarsus. The enveloping tendon sheath was identified in the horizontal sonograms as a fine anechoic area.

### Dorsomedial ultrasonography

In this region the long medial collateral ligament and the large dorsomedial pouch of the tarsocrural joint were evaluated (Fig. 1A).

**Figure 4:**
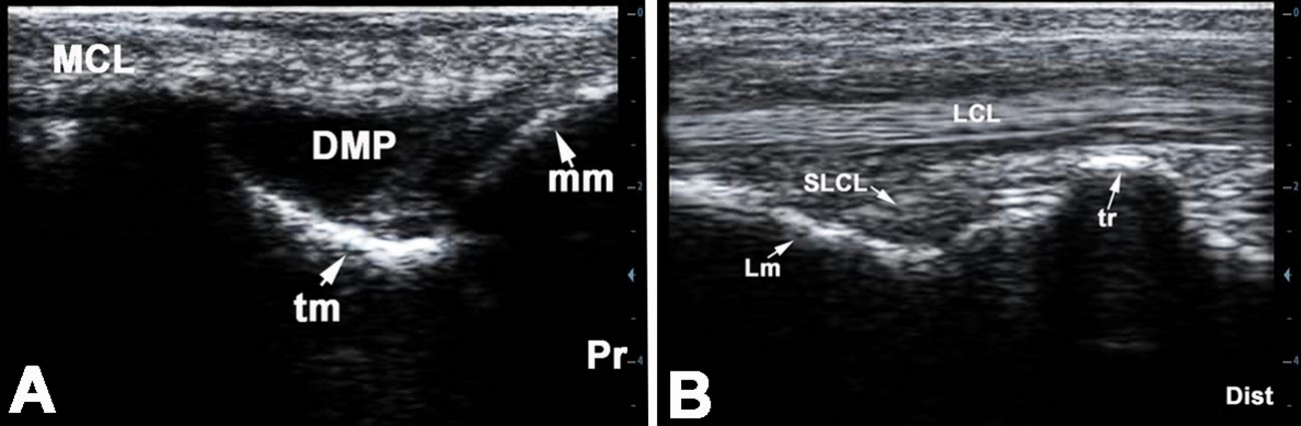
longitudinal sonogram of the medial collateral ligament at the level of the medial malleolus (**A**) and longitudinal sonogram of the lateral collateral ligament at the level of the base of the calcaneus (**B**) of the normal right tarsal region in a 7 years old male donkey. Pr, proximal; Dist, distal; DMP, dorsomedial pouch of the tarsocrural joint; mm, medial malleolus; tm, medial trochlear ridge of the talus; Lm, lateral malleolus (distal part of the fibula); tr, lateral trochlear ridge of the talus; LCL, long lateral collateral tarsal ligament; sLCL, Short lateral collateral tarsal ligament.

In level 1, only the strongly echogenic bone contour of the tibia was seen in the sagittal and horizontal sonograms. In level 2, the origin of the long medial collateral ligament from the medial tibial malleolus was seen with a sagittal transducer position and a lateroplantarly oriented sound beam and the ligament could be traced further distally (Fig. 4A). In level 3, the parallel echogenic fibers of the medial collateral ligament could be outlined over the protruding contour of the talus. In level 4, the long medial collateral ligament became flattened and attached to the medial surface of the main metatarsal bone.

On the horizontal sonograms in level 2, the ligament was depicted as an oval structure with coarse echo-pattern. A short medial collateral ligament (Ligamentum collateralia medialia brevia) could be seen between the fibers of the long medial collateral ligament and the underlying tarsal bone, which connected the directly adjacent tarsal bones with each other. In contrast, accurate imaging of the long collateral ligament at level 3 was more difficult. At this level, the ligament increasingly merged with the surrounding tissues and its image was vaguely distinguished over the medial surface of the talus.

In level 4 at the level of the distal row of tarsal bones and the metatarsus, the medial collateral ligament fanned out greatly with flat indistinct cross-section.

### Plantar ultrasonography

In this region, the insertion of the common calcaneal tendon in the calcaneal tuber, the course of the superficial (SDFT) and deep (DDFT) digital flexor tendons and their tendon sheaths and the long plantar ligament could be evaluated (Fig. 1C).

In level 1 longitudinal plane, the insertion of the common calcaneal tendon in the calcaneal tuber on one side and the course of the SDFT on the other side of the calcaneal tuber could be evaluated. The mighty contour of the calcaneal tuberosity served as a guide and inserted itself into the ultrasound image as a strongly echogenic curved line. The common calcaneal tendon was recognized by its strongly hyperechoic pattern. However, the exact lower delimitation of the tendon was very uncertain. In contrast, the clearly visible tendinous structure of the SDFT could be optimally defined in all animals. In levels 2 and 3, the SDFT and the long plantar ligament were visualized above the hyperechoic bone line of the calcaneus. The long plantar ligament was seen between the SDFT and the echo contour of the base of calcaneus and the fourth tarsal bone when the transducer was positioned exactly axially or slightly laterally.

**Figure 5:**
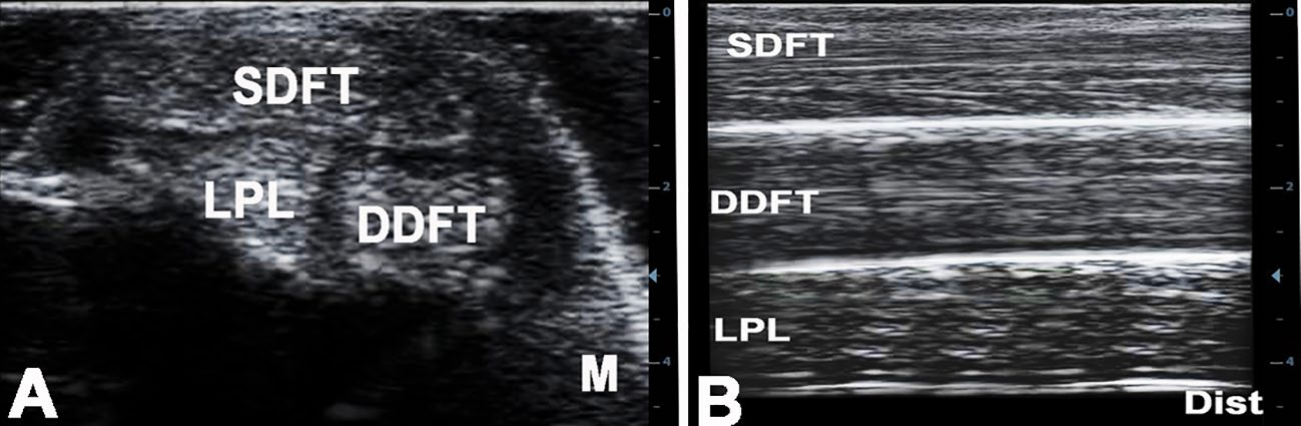
transverse (**A**) and longitudinal (**B**) ultrasonograms of the plantar aspect of the left tarsal region in a 6 years old male donkey at the level of the base of the calcaneus. M, medial; Dist, distal; SDFT, superficial digital flexor tendon; DDFT, deep digital flexor tendon; LPL, long plantar ligament.

In plane 4, the DDFT moved plantarly from the medial aspect of the tarsus far enough to be visualized lying beneath the SDFT on the plantar axial scan line. The distal portion of the accompanying tendon sheath of the DDFT was seen as an anechoic line between the two flexor tendons (SDFT and DDFT). As a result, the two tendons could be easily differentiated and outlined from one another. The echogenicity of both tendons was approximately the same in the ultrasound image (Fig. 5B).

In the horizontal plane in level 1, the SDFT was depicted as homogenous uniformly distributed strongly echogenic pin point echo-pattern and the less echogenic and irregularly patterned common calcaneal tendon underneath. At this level the SDFT tendon had an oval shape, while it widened and flattened out at the tuber calcanei. The common calcaneal tendon appeared round to oblong in shape and its edges were vaguely distinguished. In level 2, the oval shape of the SDFT was very well delimited in sonograms by its peri-tendinum, and the origin of the long plantar ligament at the tuber calcanei could be observed. The long plantar ligament had irregular shape and unclearly defined over the strongly hyperechoic contour of the calcaneus. The echo-pattern of the SDFT was stronger and more densely patterned than that of the long plantar ligament. In level 3, the three structures could be seen above the bony contour of the base of the calcaneus: the SDFT had an oval to tangerine disc shape, the long plantar ligament had irregular shape and was difficult to define, and the DDFT appeared in a rather transversely stretched oval shape. The SDFT and DDFT tendons showed equal echogenicity with a homogeneous echo-pattern. The long plantar ligament appeared echogenic with irregular outline. In level 4, it was possible to display the SDFT and DDFT tendons in the same sonogram. The insertion point of the long plantar ligament at the distal tarsal and metatarsal bones could vaguely be delineated (Fig. 5A).

### Lateroplantar ultrasonography

In this region, the lateroplantar joint pouch of the tarsocrural joint and the long lateral collateral tarsal ligament were evaluated (Fig. 1B). The horizontal and longitudinal visualization of the lateroplantar joint pouch of the tarsocrural joint was achieved by rotating the transducer head about 10–15 degree plantarly and slightly pressing the transducer against the skin in level 1. The pouch was detected as a triangular or polygonal anechoic space between the dorsal strong reflection of the tuber calcanei bone and the plantar contour of the tibia. It was not always easy to distinguish this pouch from its surroundings.

In levels 2–4 in the longitudinal plane, the long lateral collateral ligament could be well recognized, traced and demarcated from the surroundings with its parallel collagen fibers (Fig. 4B). In level 2, the ligament could be observed while passing over the hyperchoic contour of the tibia. In addition, an echo-free area lying in a bone cavity could be seen. This could be addressed as the distal portion of the lateroplantar pouch of the tarsocrural joint. In level 3, a hyperechoic line representing the base of the calcaneus was observed bulging under the ligament. In level 4, the insertion of the ligament at the lateral metatarsal bone was evaluated.

In the horizontal plane in level 2, the ligament appeared as oval structure with evenly distributed hyperechoic pin point eco-pattern over the body of the talus. However, its clear demarcation from the surroundings was not as easy as demarcating a tendon in a horizontal section. In turn, the hypoechoic to anechoic clearly definable distal portion of the lateroplantar pouch of the tarsocrural joint could be recognized. In level 3, the irregular oval appearance of the ligament on the base of calcaneus could be detected.

### Medioplantar ultrasonography

On this side, the common tendon of the lateral digital flexor and caudal tibial muscles and the medial digital flexor tendon were evaluated. The three tendons could be imaged in the same sonogram only in level 4 (Fig. 1C).

In plane 1, either the purely muscular or the muscle–tendon transition of these muscles could be displayed with a sagittal transducer position. In plane 2, the parallel collagen fibers of the common tendon of the lateral digital flexor and caudal tibial muscles were clearly delineated, since the tendon was under laid by the echogenic bone contour of the calcaneus. In level 3, the tendon was traced while progressing over the sustentaculum tali and the distal row of tarsal bones. In level 4, the transducer had to be moved further plantarly to visualize the common terminal tendon over the first and second tarsal bone.

When the transducer was held horizontally, the common tendon of the lateral digital flexor and caudal tibial muscles could be clearly visualized in plane 1 with a posteriorly tilted transducer position. In this area, the muscle–tendon transition could was seen as either inhomogeneous echo-pattern or purely tendinous tissue with a more echo-dense evenly dotted pattern. In plane 2, the tendon was bounded by the bone contour of the calcaneus. In level 3, the oval cross-section of the tendon was seen over the hyperechoic bone contour of the sustentaculum tali.

### Ultrasonography of the cunean tendon

**Figure 6:**
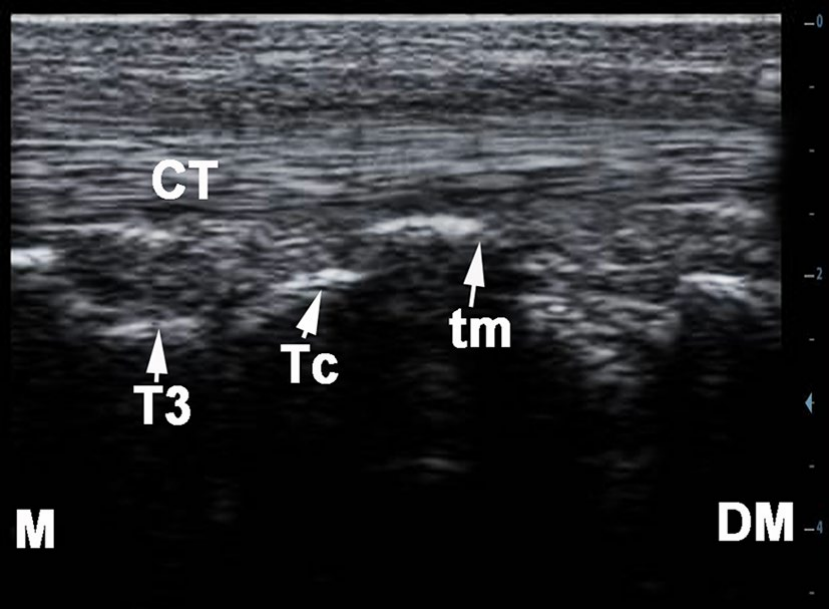
longitudinal ultrasonogram of the cunean tendon on the medial aspect of the normal right tarsal region in a 9 years old male donkey. M, medial; DM, dorsomedial; Tc, central tarsal bone; T3, third tarsal bone; tm, medial trochlear ridge of the talus; CT, cunean tendon.

For visualization of the cunean tendon, the transducer was placed in the dorsal examination line between the 2^nd^ and 3^rd^ levels. The medial trochlear ridge of the talus served as a reference point for localizing the tendon. The transducer was placed parallel to the direction of the tendon fibers and tilted medially. In this position, it was possible to follow the course of the tendon up to its insertion on the first and second tarsal bone and on the medial styloid head of the metatarsus by moving the transducer few centimeters in the medioplantar direction (Fig. 6).

## Discussion

In this study, the normal echo-graphic appearance of the tarsal region in donkeys was described; giving basic reference data for investigation of various disorders affecting the donkey tarsus. A standardized examination protocol was presented, where the tarsal region was systematically divided into 4 regions (dorsal, plantar, medial and lateral), and each region was subdivided into four levels (anatomical landmarks) in order to thoroughly investigate the numerous soft tissues constituting the donkey tarsus. Structures evaluated from the dorsum included the tendons of fibularis tertius, carnial tibial and long digital extensor muscles as well as the tarsocrural joint pouches. Medial structures included the medial collateral ligaments and the cunean tendon. Lateral structures involved the lateral digital extensor tendon and the lateral collateral ligaments. Structures depicted at the plantar aspect of the tarsus embraced the common calcaneal tendon, the long plantar ligament, the SDFT and the DDFT. The current systematic ultrasonographic protocol permitted examination of the clinically relevant structures of the donkey tarsus. Similar approaches have been previously described in horses and camels^18,22,24^.

Bones constituting the tarsus in horses include the distal end of the tibia, tarsal bones and the metatarsal bones. Tarsal bones involve the proximal or crural row (talus, calcaneus); the middle or inter-tarsal row (central tarsal bone); and the distal or metatarsal row (the fused first and second, the third and fourth tarsal bones)^21^. On sonograms, bone surfaces showed regular contours that were detected on sonograms as linear to convex hyperechoic reflections with distal acoustic shadowing. Irregular bone contours (when detected by ultrasonography) might indicate osteochondrosis, fractures, osteomyelitis, or physitis^25^. Frequently, subtle bone irregularities caused by osteomyelitis or physitis could be discovered via ultrasonography earlier than by radiography^13^.

The tarsus is a composite joint encloses multiple synovial sacs and embraces four levels of articulation^19^: the tarsocrural joint (between tibia and talus), the proximal intertarsal joint (between the talus and calcaneus proximally and the central and fourth tarsal bones distally), the distal intertarsal joint (between the central tarsal bone proximally and the small tarsal bones distally) and the tarsometatarsal joint (between distal tarsal bones proximally and the metatarsal bones distally). In the present investigation, the synovial sacs in the donkey tarsus were vaguely outlined in the longitudinal plane at the level of the joint space as an echoic to hypoechoic space. Unlike in horses,^18^ a clear demarcation between the joint capsule and the surrounding soft tissues was only possible at the level of the tarsocrural joint in the longitudinal plane where the joint capsule was more echogenic and could be clearly outlined. Furthermore, investigation of joint sacs of the intertarsal and the tarsometatarsal joints was a challenge, as previously described in horses, camels and dogs^18,22–24^.

In the present study, differentiation and the demarcation of ligaments, muscles, tendons and the muscle–tendon transition was very well possible and could be easily followed during their course over the donkey tarsus. Tendons and ligaments were recognized and assessed on the basis of the alignment of their parallel collagen fiber bundles in the longitudinal plane and the uniformly echogenic dot pattern in the horizontal image. Similar criteria have been described in horses, camels and dogs^18,22–24^. Interestingly in the donkey tarsus, complete evaluation of some soft tissue structures required certain technical skill. The transducer should be tilted 15^o^ laterally for an optimum longitudinal scanning of the long digital extensor tendon; pressing of the transducer against the skin to evaluate the cranial tibial vein; moving the transducer head medial to the axial line of the tarsus to visualize the medial pouch of the tarsocrural joint; and rotating the trancducer head plantarly about 10–15 degree with slight pressure to assess the laterplantar joint pouch of the tarsocrural joint.

The medial and lateral tarsal collateral ligaments arise from the ipsilateral tibial malleolus, attach to tarsal bones along their course and terminate in the metatarsal bone. Each collateral ligament is an aggregation of deep short ligaments and a long superficial ligament. The short lateral collateral ligaments run beneath the long lateral collateral ligament and attach to the talus and calcaneus. The short medial collateral ligaments attach to the medial aspect of the talus^26^. In the present investigation, the long collateral ligament was easily identifiable in the longitudinal sonograms by its densely packed linear fiber pattern; however, the examiner should follow the ligament from its origin to insertion to confirm imaging of the long lateral collateral ligament to be differentiated from the adjacent lateral digital extensor tendon. Only one medial and on lateral short collateral ligaments could be recognized in the current study, the short collateral brevis ligament. Other short components of the collateral ligaments were challenging to visualize and couldn’t be recognized by ultrasound, may be due to their small size and their course and curving over the bony prominences of the talus. Similar outcomes were recorded in horses and camels^18,23,24^.

In this study the SDFT was flattened, had crescent shape at the calcaneal tuber. It continued distally through the tarsus and metatarsus along the plantar midline. The DDFT was located plantaromedially throughout the tarsal region and coursed medial to the calcaneus. The long plantar ligament was located along the plantarolateral aspect of the tarsus. Both the SDFT and DDFT had similar echogenicity. Differentiation of tendons from one another relies on the anatomical location (the DDFT is deeper to the SDFT) and differentiation of both tendons from one another depends on detection of the tendon sheath of the DDFT, which was recognized as an anechoic line between the SDFT and DDFT.

On the base of these premises, a methodological and descriptive protocol for the echo-graphic examination of the tarsal region in donkeys has been proposed. The easily identifiable anatomical landmarks and the subdivision of the tarsus into four regions and four levels were useful aids for orientation and allowed thorough ultrasonographic investigation of the donkey tarsus.

## Methods

The present study was permitted by the Research Ethics Committee (REC) of King Faisal University, KSA (Permit Number: KFU-REC/2022-03-11). All procedures were carried out in accordance with the relevant guidelines and regulations. The methods were reported in accordance with ARRIVE guidelines and regulations.

### Animals

Based on previous similar studies,^18,22,27^ the present study included 11 adult clinically sound donkeys (6 males and 5 non pregnant females), aging 6.5 ± 3.2 years (age range 5–9 years) and weighing 140 ± 32 kg (115–150 kg). Animals were apparently healthy based on orthopedic and clinical examinations and exempt from hind limb lameness or tarsal pathologies. Animals were kept in the Veterinary Teaching Hospital, College of Veterinary Medicine, King Faisal University, Kingdom Saudi Arabia for 15 days prior to the study. Animals were accommodated and cared for according to the Saudi animal law (Ministry of Environment, Water and Agriculture; No. 39, 2013). Food and water were introduced ad libitum. By the end of the study donkeys were returned to the farm of the College of Veterinary Medicine, King Faisal University. Five donkey cadaveric tarsi were obtained from 3 donkey cadavers (2 males and one female, aged 7 ± 1 years and weighted 150 ± 15 kg), collected from the department of pathology, College of Veterinary Medicine, King Faisal University, that were euthanized for reasons unrelated to study. Cadaveric tarsi were dissected to explore the anatomy of the donkey tarsal region.

Prior to ultrasonography, the left and right tarsi of each donkey were clipped with #40 blades, washed with warm water and soap and ultrasound coupling gel was applied. Standoff pad was not used in the current study. All examinations were performed while the animal was in the standing position. During examination sedatives were not required and sometimes physical restraining was used.

### Ultrasonographic technique

Ultrasonographic exploration of the tarsal joint was performed using an ultrasound machine (ProSound 3500SX, Osaka, Japan) supplied with a multi-frequency (5–12 MHz) linear transducer (“T” probe).

The donkey tarsal region was divided into four regions protocol (dorsal, medial, lateral, and plantar). Systematic ultrasonographic investigation of each region was performed at four horizontal levels (anatomical landmarks) in each region. The levels were at the calcaneal tuber (level 1), tibial malleoli (level 2), base of the calcaneus (level 3) and at the proximal end of the metatarsal bone (level 4). Ultrasonography of every region at each level was performed in both the longitudinal and transverse planes.

## Data Availability

The datasets used and/or analyzed during the current study are available from the corresponding author on reasonable request.
